# RNA *in situ* hybridization of *Escherichia coli* in equine endometrial biopsies

**DOI:** 10.3389/fvets.2023.1186051

**Published:** 2023-06-09

**Authors:** Elina Tukia, Isa Hallman, Maarit Penttilä, Satu Hänninen, Maria Kareskoski

**Affiliations:** ^1^Department of Production Animal Medicine, Faculty of Veterinary Medicine, University of Saarentaus, Saarbrücken, Finland; ^2^Department of Pathology, Faculty of Medicine, University of Helsinki, Helsinki, Finland

**Keywords:** mare, endometritis, *Escherichia coli*, dormant bacteria, biofilm

## Abstract

Endometritis is one of the major causes of infertility in mares. *Escherichia coli* and *β*-haemolytic streptococci are among the bacterial species most frequently isolated from the equine uterus. Some bacteria such as *β*-hemolytic streptococci, can persist in dormant forms and cause prolonged, latent or recurrent infections. Dormant bacteria may be present despite negative bacterial cultures, and they are resistant to antimicrobial treatment due to their resting metabolic state. The purpose of this study was to study formalin-fixed paraffin-embedded equine endometrial biopsies for the presence and localization of *E. coli*—bacteria, with a chromogenic RNAscope^®^-method for detection of *E. coli*-related 16S ribosomal RNA. Hematoxylin-eosin—stained endometrial biopsies were evaluated to determine the level of inflammation and degeneration. During estrus, samples were taken for endometrial culture and cytology with a double-guarded uterine swab. The samples included eight samples with moderate to severe endometrial inflammation detected in endometrial histopathology, and growth of *E. coli* in bacterial culture, six samples with moderate to severe endometrial inflammation but negative bacterial culture, and five samples with no endometrial pathology (grade I endometrial biopsy, negative endometrial culture and cytology) serving as controls. Positive and negative control probes were included in the RNA *in situ* hybridization, and results were confirmed with a fluorescence detection method (fluorescence *in situ* hybridization). Only unspecific signals of limited size and frequency of occurrence were detected in all samples, with random localization in the endometrium. No samples contained rod-shaped signals corresponding to bacterial findings. In conclusion, there was no evidence of bacterial invasion in the endometrium regardless of the inflammatory status of the biopsy or previous bacterial culture results. According to these findings on a small number of samples, invasion of *E. coli* is not a common finding in the lamina propria of mares, but these bacteria may also evade detection due to localized foci of infections, or supra-epithelial localization under the cover of biofilm. These bacteria and biofilm covering the epithelium may also be lost during formalin-fixation and processing.

## 1. Introduction

Endometritis is one of the major causes of subfertility in mares (1, 2). Post-mating endometritis is a normal physiological reaction to semen in the uterus, and most mares are able to restrict it, with a well-regulated, normal inflammatory reaction and effective uterine contractions (2–4). Some mares are more susceptible to bacterial endometritis because of impaired uterine physical clearance that is caused by anatomical and degenerative defects (2). Mare age, repeated endometrial inflammation, breeding, multiple pregnancies, and parturition are potential factors causing endometrosis, a progressive and irreversible fibrotic condition of the endometrium. Recurrent infections may also contribute to the development of degeneration and fibrosis, which can, in turn, also make the endometrium more prone to adhesion of *Streptococcus equi* subsp*. zooepidemicus—*bacteria (5).

The diagnosis of endometritis includes both clinical examination and diagnostic laboratory tests, such as cytology, culture, and histopathology (6). Histopathology is the golden standard for the diagnosis of endometritis, especially in mares suffering from subclinical endometritis (6, 7). The most common pathogenic bacteria isolated are *β*-hemolytic streptococci and *E. coli* (7, 8) but in a recent study by Teixeira-Soares et al. (6), *Staphylococcus* spp. was found more frequently than *Streptococcus* spp. *Streptococcus equi* subsp. *zooepidemicus* has been shown to cause subclinical, dormant infections in the endometrium, with dormant bacteria re-activated after intrauterine inoculation with a bacterial growth medium. This kind of persistent bacterial cells tolerate antibiotic treatment and other stress factors better than normal growing cells, which makes treatment even more challenging (9).

Endometritis may be associated with prolonged, latent or recurrent infections with bacteria such as persistent dormant *β*-hemolytic streptococci (9). Dormant bacteria may be present despite negative bacterial cultures, and they are resistant to antimicrobial treatment due to their resting metabolic state. In other species, *E. coli* has been known to develop certain adhesion factors that enable persistence in the genitourinary tract, causing recurring clinical inflammation. In humans, uropathogenic *E. coli* is known to cause recurrent urinary tract infections. In these cases, dormant intracellular *E. coli* has been shown to be re-activated by normal vaginal microbiota, such as *Gardnerella vaginalis* (10). Uropathogenic *E. coli* is also the most commonly isolated pathogen in canine pyometra (11, 12) and similarity between *E. coli* virulence factor genes in canine pyometra and human recurrent urinary tract infections has been found (11, 13). The binding of bacteria to endometrial cells requires adhesins, which are encoded mainly by three adhesin genes (fimH, papGIII and sfa). Type 1 fimbriae (FimH) mediates binding of *E. coli* to erythrocytes, leukocytes, and uroepithelial cells (12–14).

It is not well established whether persistent or latent *E. coli* –invasion occurs in the equine endometrium. By using *in situ* hybridization, Jäger et al. (8) found *E. coli* RNA in endometrial biopsies in only 4% of *E. coli* culture positive mares (*n* = 99). In their study, 36% of the *E. coli* culture positive mares had detectable endometrial inflammation in the uterine biopsy, and no inflammation was detected in the mares in which *E. coli* could be demonstrated with *in situ* hybridization. Buczkowska et al. (7) compared endometrial cytology and endometrial biopsy as diagnostic methods for endometritis, and found that only one in four mares with *E. coli* positive bacterial cultures of biopsies had a positive cytology, confirming that *E. coli* may be present without inducing any significant endometrial inflammatory reaction.

The objective of this study was to determine if the presence of *E. coli* in the endometrium can be demonstrated with RNA *in situ* hybridization, indicating a latent endometrial infection similar to the uropathogenic and dormant bacteria in humans and dogs. To this end, formalin-fixed paraffin-embedded equine endometrial biopsies were studied for the presence and localization of *E. coli*-bacteria, with a chromogenic RNAscope^®^-method for detection of *E. coli* 16S ribosomal RNA.

## 2. Materials and methods

### 2.1. Animals

A total of 19 mares residing at various locations were included in the study. The age of the mares ranged from eight to 20 years [mean age 14.2 (8–20) years]. The breeds of the mares included Finnhorses (*n* = 6), Standardbred trotters (*n* = 3), Warmblood sport horses (*n* = 8), one Friesian mare, and one Shetland pony. The history of the mares was varied, with mares classified as maiden (*n* = 3), foaling (*n* = 6), open (not bred) (*n* = 4), or barren (*n* = 6), and parity before sample collection was 1–6 (median = 2). The breeding and foaling data of the mares were recorded until the end of the year 2022 (1–11 years).

### 2.2. Samples

The samples were classified in three groups according to uterine culture results and severity of inflammation: (1) eight samples with moderate or severe endometrial inflammation observed in endometrial histology, in combination with growth of *E. coli* in previous bacterial culture; (2) six samples with moderate or severe endometrial inflammation but negative bacterial culture; and (3) five samples with no endometrial pathology. The samples without pathology were deemed healthy controls.

Endometrial samples submitted to the Veterinary Teaching Hospital at the University of Helsinki, Finland, for evaluation of endometrial pathology in clinical cases were used in the study. All samples were taken by different referring veterinarians, and they were accompanied by an information form containing the relevant medical history of each mare. The samples were collected during the breeding season in the estrous phase. After transrectal palpation and ultrasonography, the mare’s tail was wrapped and the perineal area was washed three times with disinfectant soap, rinsed with water, and dried with paper towels. The endometrial biopsies were taken from the base of one uterine horn, using biopsy alligator jaw forceps. The biopsy was immediately placed into 10% formalin and submitted to the laboratory for processing. Hematoxylin-eosin—stained endometrial biopsies were evaluated to determine the level of inflammation and degeneration. The formalin-fixed endometrium was embedded in paraffin and sections were stained with hematoxylin (H9627, Sigma-Aldrich, Darmstadt, Germany) and eosin (HT1103128; Sigma-Aldrich, Darmstadt, Germany).

The endometrial biopsies were examined under a light microscope, and graded based on the extent of inflammation and/or degeneration, according to the Kenney and Doig classification (15). Category I included samples with normal endometrium or only mild and sparse inflammation or fibrosis, category IIA included samples with mild and scattered inflammation and fibrosis, category IIB included samples with moderate inflammation or fibrosis, and category III were characterized by severe irreversible fibrosis and/or inflammation.

Samples were also obtained for bacterial culture and endometrial cytology, and these samples were taken before the endometrial biopsy. A similar perineal wash as described earlier was done before sample collection. The veterinarian, wearing a clean rectal sleeve turned inside out, and a sterile glove on the sample collecting hand, obtained the samples for culture and cytology using a double-guarded uterine culture swab (Minitube, Tiefenbach, Germany, or Equivet, Langeskov, Denmark, according to practitioner preference and availability). The samples were submitted to the laboratory for culture in transport culture media (various manufacturers). For uterine cytology, samples were gently rolled on glass microscope slides and air-dried.

On arrival in the laboratory at the Veterinary Teaching Hospital, the cytology slides were stained using the Diff Quick stain (manufacturer), air-dried, and examined with a light microscope (Olympus BX40; Olympus America, Center Valley, PA, United States). Slides were initially evaluated using a 10x objective (100× magnification), and the areas populated by cells were examined with a 100× objective (1,000× magnification) under oil. For bacterial culture, the swab was removed from the transport medium, cultured on a sheep blood agar and incubated at 37°C in atmospheric air. The number of colony-forming units on the plate was counted after 24 h of incubation. If no growth was observed, incubation was continued for another 24 h. Bacterial colonies present at 24 or 48 h after incubation were evaluated using light microscopy and Gram-staining. Gram-negative bacteria were cultured on MacConkey agars and incubated for 24 h. Samples with a minimum of 5 to 10 colonies of gram-negative bacteria exhibiting a typical colony appearance with pink coloration due to lactose fermentation were determined to be positive for *E. coli.*

### 2.3. RNA *in situ* hybridization

RNA *in situ* hybridization was performed on fresh 5 μm formalin-fixed paraffin embedded (FFPE) tissue sections using RNAscope 2.5 HD Reagent Kit-RED (#322350, Advanced Cell Diagnostics, Bio-techne, Newark, United States) for target detection according to the manual. For positive control, the commercial *E. coli* strain JM109 (Promega Corporation, Wisconsin, United States) embedded in agarose, was formalin-fixed, paraffin embedded, and cut to 5 μm sections. In brief, tissue sections were baked for 1 h at 60°C, then deparaffinized and treated with hydrogen peroxide for 10 min at room temperature. Target retrieval was performed for 15 min at 98°C, followed by protease plus treatment for 15 min at 40°C. The biopsies were stained with an *in situ* hybridization (ISH) probe targeting *E. coli* RNA. The *E. coli* probe (RNAscope technology, Probe B-E.Coli-16SrRNA cat# 433291, Advanced Cell Diagnostics, Newark, CA), positive control Ec-PPIB (#462351), and negative control DapB (#310043) were hybridized for 2 h at 40°C followed by signal amplification steps. The samples were incubated for 50 min with AMP 5–reagent. The samples were then treated with fast red for 10 min at room temperature followed by counterstaining with 50% hematoxylin. The sections were dipped in 0.02% ammonium water and dehydrated for 15 min at +60°C before mounting with EcoMount (EM897L, Biocare Medical). Tissue sections were scanned using 3DHISTECH Panoramic 250 FLASH III digital slide scanner using 1× 40 magnification with extended focus and 7 focus levels at Helsinki Biobank supported by Hospital District of Helsinki and Uusimaa (HUS), the University of Helsinki, Kymenlaakso Social and Health Services (Carea) and the South Karelia Social and Health Care District (Eksote).

### 2.4. Statistical analyses

Due the observational nature of the study and the small sample size, only frequencies and descriptive data were calculated, and reported as mean values ± SE mean (min–max). All calculations were done on IBM SPSS Statistics version 29.0.0.0 (IBM SPSS, Chicago, Illinois, United States).

## 3. Results

### 3.1. Descriptive data

Data on mare history, breedings, and pregnancy outcome after sample collection (endometrial biopsy) in groups 1, 2, and 3 are shown in Table 1, and in mares with different endometrial biopsy gradings (grade I, IIA, IIB, or III) in Table 2.

**Table 1:** Data on mare history, breeding opportunity and pregnancy outcome after sample collection (endometrial biopsy) in group 1 (samples with moderate or severe endometrial inflammation observed in endometrial histology, in combination with growth of *Escherichia coli* in previous bacterial culture), group 2 (samples with moderate or severe endometrial inflammation but negative bacterial culture), and group 3 (samples with no endometrial pathology).

	Group 1 (*n* = 8)	Group 2 (*n* = 6)	Group 3 (*n* = 5)
*Mare history*			
Mare age at biopsy (years)	14.5 ± 1.1 (10–19)	14.2 ± 2.0 (9–20)	13.8 ± 2.1 (8–19)
Mare status at biopsy	2 maiden 4 foaling 1 open 1 barren	1 maiden 2 foaling 1 open 2 barren	0 maiden 0 foaling 2 open 3 barren
Number of live foals before biopsy	2.5 ± 0.9 (0–6)	0.8 ± 0.6 (0–4)	1.4 ± 0.4 (1–3)
Number of years open (not bred) before biopsy	0 years (7 mares) 3 years (1 mare)	0 years (4 mares) 8 years (1 mare) 11 years (1 mare)	1 year (2 mares) 2 years (2 mares) 3 years (1 mare)
Number of years barren before biopsy	0–1	0–3	0–1
*Breedings and pregnancy outcome*			
Number of mares bred after biopsy/total number of mares	4/8	0/6	5/5
Number of estrous cycles bred after biopsy	1–4	0	2–5
Number of years open (not bred) after biopsy until next breeding	0–1 (6 mares) 2 mares not bred again	0/6 mares bred again	0/5 mares bred again
Number of years barren after biopsy until next pregnancy	0–1 (6 mares) 2 mares not bred again	0/6 mares bred again	0/5 mares
Number of mares pregnant after biopsy	4/8	0/6	2 unknown 3 not pregnant
Number of foals after biopsy (up to 2022)	0 foals: 4 mares 1–2 foals: 4 mares	0 foals	1 foal

Data presented as mean ± SE (min-max) where applicable.

**Table 2:** Biopsy grades, experiment groups (group 1: samples with moderate or severe endometrial inflammation observed in endometrial histology, in combination with growth of *Escherichia coli* in previous bacterial culture); (group 2: samples with moderate or severe endometrial inflammation but negative bacterial culture); and (group 3: samples with no endometrial pathology), and data on mare history, breeding opportunity and pregnancy outcome after sample collection (endometrial biopsy).

	Biopsy grade				All mares (*n* = 19)
	I (*n* = 2)	IIA (*n* = 6)	IIB (*n* = 10)	III (*n* = 1)	
*Sample group*					
Group 1	0 mares	2 mares	5 mares	1 mare	8 mares
Group 2	0 mares	1 mare	5 mares		6 mares
Group 3	2 mares	3 mares			5 mares
*Mare history*					
Mare age at biopsy (years)	8 and 11	14.0 ± 1.7 (9–19)	15.1 ± 1.2 (9–20)	16	14.2 ± 0.9 (8–20)
Mare status at biopsy	2 open	1 maiden 2 foaling 3 barren	1 maiden 4 foaling 2 open 3 barren	1 maiden	3 maiden 6 foaling 4 open 6 barren
Number of live foals before biopsy	0 foals: 0 mares 1–3 foals: 2 mares 4–6 foals: 9 mares	0 foals: 0 mares 1–3 foals: 4 mares 4–6 foals: 1 mare	0 foals: 3 mares 1–3 foals: 5 mares 4–6 mares	0 foals: 1 mare 1–3 foals: 0 mares 4–6 foals: 0 mares	0 foals: 3 mares 1–3 foals: 12 mares 4–6 foals: 4 mares
Number of years open (not bred) before biopsy	1 and 3	1.0 ± 0.5 (0–2)	2.4 ± 1.4 (0–11)	0	1.9 ± 0.8 (0–11)
Number of years barren before biopsy	0	0.7 ± 0.2 (0–1)	0.6 ± 0.3 (0–3)	1	0.6 ± 0.2 (0–3)
*Breedings and pregnancy outcome*					
Number of mares bred after biopsy/total number of mares	2/2	5/6 mares	4/10 mares		11/19
Number of estrous cycles bred after biopsy	2 and 3	2.8 ± 0.7 (0–5)	0.6 ± 0.3 (0–2)	0	1.47 ± 0.4 (0–5)
Number of years open (not bred) after biopsy until next breeding	1 and 3	0–2	0–11 years (6/10 mares with 0 years open)	1	0–1
Number of years barren after biopsy until next pregnancy	0	0–1	0–1	1	0–1
Number of mares pregnant after biopsy	2 unknown	2/6 mares	2/10 mares	0/1 mares	4/19 mares
Number of foals after biopsy (up to 2022)	0	0.5 ± 0.2 (0–1)	0.3 ± 0.2 (0–2)	0	0–2

Data presented as mean ± SE (min-max) where applicable.

#### 3.1.1. History before biopsy

Before the biopsy, 84.2% of the mares had foaled at least once, with 52.7% of mares having produced one or two foals, and 31.7% of mares having produced more foals (four to seven foals). Mares had been open (not bred) for 0–11 years, and most mares (52.6%) had been bred earlier during the same breeding season, or in the previous 1–2 years (42.1% of mares).

#### 3.1.2. Breedings and pregnancy outcomes after biopsy

In 47.4% of mares, breeding was not attempted after the uterine biopsy, and the rest (11 mares) were inseminated in one, two, or three cycles (10.5, 21.2, and 15.8%, respectively). A total of six foals were born after biopsy in this population, with 73.7% of mares never foaling again after the biopsy.

#### 3.1.3. Biopsy results and pregnancy outcomes

Most of the mares (7/8 mares) with a biopsy grade of I or IIA were bred again after the biopsy was taken, whereas only 4/11 mares with a biopsy grade of IIB or III were bred after biopsy.

### 3.2. RNA *in situ* hybridization

**Figure 1:**
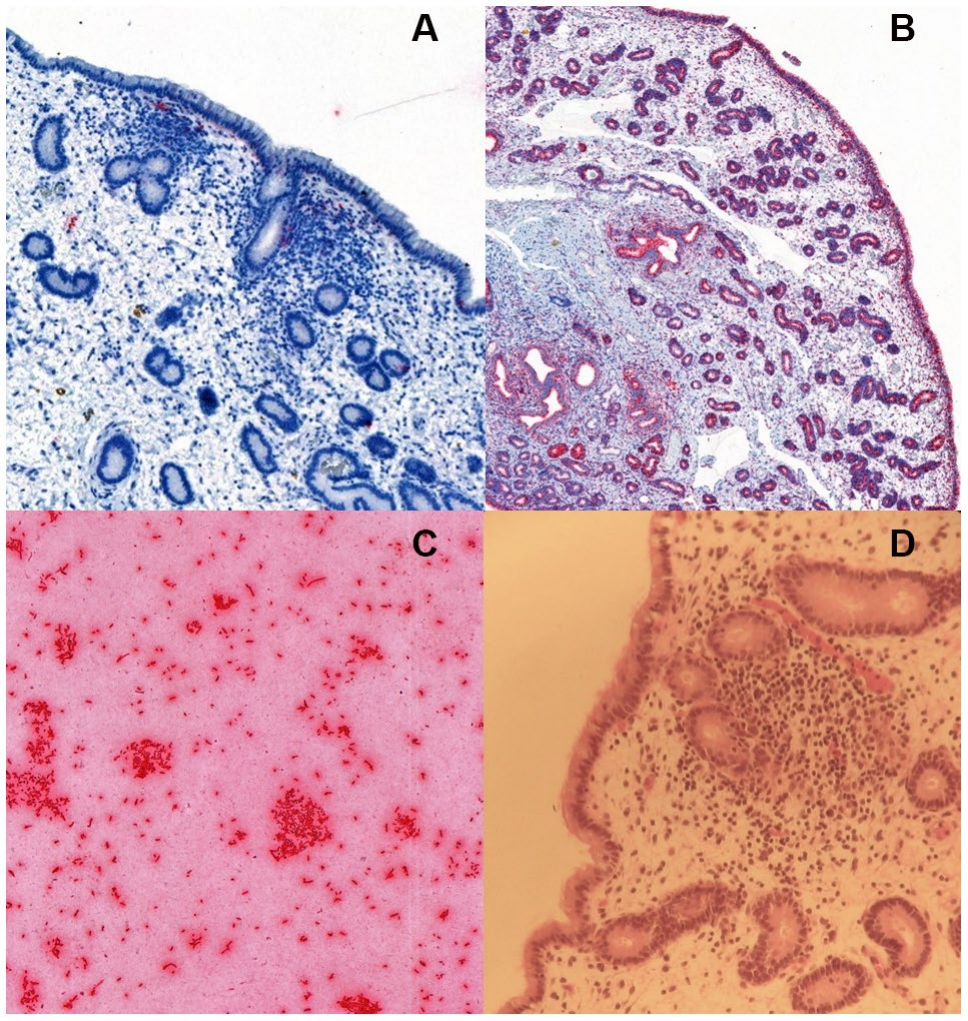
RNA *in situ* hybridization for *Escherichia coli* in **(A)** an equine endometrial biopsy sample with moderate endometrial inflammation, positive for *E. coli* in bacterial culture. The sparse and unspecific signals (detected in red colour) were considered unrelated to bacterial colonization, **(B)** a positive control with the ubiquitous housekeeping gene probe PPIB (Advanced Cell Diagnostics, Newark, CA), and **(C)** a positive control consisting of a commercial *E. coli* strain JM109 (Promega Corporation, Wisconsin, United States). The image in **(D)** shows the hematoxylin–eosin stained slide of the endometrial biopsy in **(A)**.

Unspecific signals of limited size and frequency of occurrence were detected in all samples, with random localization in the endometrium. None of the samples contained any rod-shaped signals that could be attributable to the presence of *E. coli* in the lamina propria of the endometrium, endometrial glands or covering the endometrial epithelium (Figure 1).

## 4. Discussion

In the present study, *E. coli* could not be detected from the endometrium with RNA *in situ* hybridization. *E. coli* is known for the ability to produce persistent and recurring infections through biofilm formation, especially in the urinary tract (16), but persistence in the equine endometrium has not been documented to this date. The mechanisms of bacterial persistence in the endometrium other than biofilm production are not well described in the horse. In a report on persistent streptococcal infections Petersen et al. (9) described dormant forms of the bacteria able to resist antibiotic treatment, return to an active state after uterine inoculation with a growth enhancing media, resulting in clinical endometritis. Streptococci have also been documented to survive intracellularly in *in vitro* cultures (16), but the mechanism of persistence may differ from that of *E. coli*. Some *E. coli* strains can replicate intracellularly (17) but it is not known whether this occurs in the equine endometrium. The healthy uterus harbors a core uterine microbiome of which bacteria of the *Escherichia* genus are a part, together with *Lactobacillus, Shigella, Streptococcus, Blautia, Staphylococcus, Klebsiella, Acinetobacter*, and *Peptoanaerobacter* (18). Persistent or recurrent infections are likely associated with mare-dependent factors, such as altered uterine immunity, or anatomical or functional defects (2).

A study by Jäger et al. (8) studied bacterial colonization of the equine endometrium with ISH, and detected streptococci and *E. coli* in the luminal epithelium and the superficial secretory ducts of the uterine glands. Biofilm plaques were not reported. All endometrial samples in the present study were fixed with a formalin fixative. In previous reports, formaldehyde has been described as inferior to Bouin’s solution as the latter preserves mucus layers and bacterial biofilms in the samples, whereas formalin effectively removes them (19). The use of Bouin’s solution for fixation of endometrial biopsies has been commonly discontinued due to the health hazards and explosive nature of one of its compounds, picric acid (20). It is plausible that any pre-existing biofilm in the samples in our study may have been destroyed during the sample preparation and fixation process, although polymorphonuclear cells, cell debris, and secretions are commonly observed in glandular ducts, despite formalin fixation. A recent report by Viitanen et al. (21) on follicular cystitis in dogs, revealed intramural *E. coli* in dogs with a history of recurrent urinary tract infections. The location of bacteria varied, with most of the findings located in follicles of lymphoid tissue in the lamina propria, but bacteria were also detected in the epithelium and submucosa of the urinary bladder. It is unclear whether the lack of *E. coli* findings in our study is related to removal of bacteria during processing, differences between bacterial colonization of the urinary tract and endometrium, differences between host species, or the selection and low number of cases.

Jäger et al. (8) detected *E. coli* with ISH from formalin fixed endometrial samples, but the bacteria were found only in 4% of samples with a positive culture result. It has also been shown that reliable ISH results will require a density of pathogens in the primary material of at least 100,000 colony-forming units/mL (22). It is possible that the number of bacteria in our samples was not high enough for proper detection with ISH due to biofilm degradation during fixation. Additionally, Jäger et al. (8) used a different probe for *E. coli* detection, with 23S rRNA as the target rRNA (sequence: 5′-GCA TAA GCG TCG CTG CCG-3′), which does not target all *E. coli* strains (23), perhaps explaining the low prevalence in that study. To the authors’ knowledge, there are no previous reports on diagnosing the bacteria from equine endometrium with the probe used in the present study. The positive controls did, however, display clear signals.

*E. coli* is a common causative organism in chronic equine endometritis, but its persistence or recurrence may be more related to reinfection or biofilm protection than intracellular or intraglandular dormancy. In biofilms, bacterial colonies are embedded in a matrix of extracellular polymeric substances that protect the microbes from adverse environmental conditions, give them increased resistance to innate immune responses and antibiotics, and enable them to survive long periods in unsuitable and stressful environments (16, 24). When environmental conditions improve, the biofilm-protected microbes can revert to their planktonic state and be released from the biofilm, colonizing new surfaces (24). By staying dormant and hidden from the immune system, *E. coli* may cause local tissue damage and later cause an acute infection (25).

Biofilm-associated infections are typically difficult to diagnose and treat, and the rates of recurrence are high. Systemic or local antibiotics are often ineffective against bacteria residing within a biofilm, and therapy has to include biofilm disruptors in combination with antibiotics (24). Bacteria in a biofilm is 10–1,000 times more resistant to antibiotic treatment than planktonic bacteria. Biofilm increases resistance to antimicrobial treatment by physically reducing penetration of the drug and by reducing the metabolism of bacteria (26), as antibiotics only act against metabolically active bacteria. Bacteria deep within the biofilm survive and become a nidus for recurrence and development of a chronic infection (27). Antibiotic resistance genes can be rapidly transferred through horizontal gene transfer, and this has been shown to occur more frequently in biofilms than in planktonic cultures (28).

Obtaining definitive results on the presence of a uterine infection is challenging, and the studied samples may contain false positive cultures, a commonly encountered issue in endometritis diagnostics. Positive bacterial culture, together with detection of polymorphonuclear cells (PMNs), has traditionally been considered to confirm the diagnosis of endometritis (1, 29). Katila (30) reviewed the sensitivity and specificity of the diagnostic methods of endometritis, including culture swabs, cytobrush, low volume lavage, and uterine biopsy. Microbial culture is necessary to confirm a diagnosis of infectious endometritis, and low volume lavage is the most sensitive test for diagnosis of bacterial endometritis, as the fluid spreads over a large endometrial surface. A cytobrush or a swab is in contact only with a small surface of the uterus, and local inflammatory foci may remain undetected. False negative cultures may be due to a failure to recover the organisms present. The sensitivity of the different sampling methods increases if bacterial culture and cytology are done in conjunction (29, 30). The evaluation of endometrial cytology is based on the presence of PMNs indicating an active inflammation (29). *E. coli* infection seems to suppress or limit the PMN response, and *E. coli* is less likely to be associated with a positive cytology than *Streptococcus* spp., *Staphylococcus* spp., or *Klebsiella* spp. (31), and it has been suggested that some *E. coli* strains have a low chemotactic potential (32).

The samples in our study were taken with a double-guarded swabs or cytobrush by various equine practitioners, and false positives are hence possible. Uterine sampling is challenging because of the risk of sample contamination with potential pathogens from the external genitalia and vagina. Double-guarded techniques and aseptic procedures are essential to avoid false positive cultures. Positive bacterial culture should be supported by the presence of PMNs on a cytology for confirmation of the diagnosis, but variations in the generated immune response by different bacterial species and strains makes it difficult for the practitioner to draw definitive conclusions in all cases (29, 30).

The virulence factors of *E. coli* in equine endometritis have not been established. Uropathogenic *E. coli* in human samples display several virulence factors, such as adhesins, toxins, iron-acquisition systems, and immune evasion mechanisms (33). In human urinary tract infections, the prevalence of different operons coding for virulence factors has been studied using PCR. Bacterial attachment to host cells is commonly mediated by fimbriae, and according to human studies, genes encoding type 1 fimbriae (fimH) are found in the most common pathogenic strains and are considered a pre-requisite for infection (33, 34). Krekeler et al. (13) confirmed that fimH virulence factor is the most important factor facilitating binding of uropathogenic *E. coli* to canine endometrium, although other adhesins, such as papGIII and sfa, are frequently detected in *E. coli* strains from canine pyometra cases (11). Screening of *E. coli* strains and their virulence factors associated with equine endometritis by PCR for the prevalence of virulence genes could be of interest in future research.

Regarding the biopsy results and pregnancy outcomes, this data set is obviously limited in numbers and definitive conclusions cannot be drawn based on this study. After endometrial biopsy, 52.6% (11 mares) of mare population were bred in one, two or three cycles each. Only six (five live foals, one stillborn) foals from a total of 19 mares were born after uterine biopsies were taken, with 74% of mares never foaling again, reflecting perhaps the history of subfertility (i.e., the reason for taking the biopsy in the first place), and the decisions made by owners based on the biopsy results. The foals were born from mares in the categories IIA (3 foals) and IIB (2 live foals, 1 stillborn). Two mares from category I were still pregnant at the time of writing. Most of the mares (7/8 mares) with a biopsy grade or I or IIA were bred again after the uterine biopsy, whereas only 4/11 mares with a biopsy grade of IIB or III were bred after biopsy. The biopsy grade of mare tends to guide the owner’s breeding decisions, and mares with a biopsy grade of IIB and III are less likely to be bred again, than those with a biopsy grade I or IIA. When a decision is made to breed the mare, providing adequate breeding opportunity is one of the most important ways to improve results, especially when cooled, shipped semen is used, because mares bred in only one estrus have significantly lower foaling rates than mares bred in two or more cycles (35).

In conclusion, there was no evidence of bacterial invasion in the endometrium regardless of the inflammatory status of the biopsy or previous bacterial culture results. This study enrolled only a small number of animals, and invasion into the lamina propria by *E. coli* was not detected. According to these findings, invasion of *E. coli* is not a common finding in the lamina propria, but these bacteria may also evade detection by inhabiting in localized foci of infections, or by supra-epithelial localization under the cover of biofilm. These bacteria and biofilm covering the epithelium may also be lost during formalin fixation and processing, but no intramural bacteria were detected either.

## Data availability statement

The original contributions presented in the study are included in the article/supplementary material, further inquiries can be directed to the corresponding author.

## Ethics statement

Ethical approval was not required for this study because of the use of leftover archived samples collected from animals examined for breeding or subfertility. The Finnish legislation (Act on the Protection of Animals Used for Scientific or Educational Purposes 497/2013) does not require the study to be reviewed or approved by an ethics committee because no samples were collected from animals specifically for this study.

## Funding

The study was funded by the Finnish Veterinary Foundation.

## Author contributions

MK: study conception and design. MK, SH, and ET: data collection, analysis and interpretation of results. MP, IH, ET, and MK: draft manuscript preparation. All authors contributed to the article and approved the submitted version.

## Conflict of interest

The authors declare that the research was conducted in the absence of any commercial or financial relationships that could be construed as a potential conflict of interest.

## Publisher’s note

All claims expressed in this article are solely those of the authors and do not necessarily represent those of their affiliated organizations, or those of the publisher, the editors and the reviewers. Any product that may be evaluated in this article, or claim that may be made by its manufacturer, is not guaranteed or endorsed by the publisher.
